# Nonlinear associations between physical activity, sleep quality, and psychological distress in university students: a cross-sectional interpretable machine learning study

**DOI:** 10.3389/fpubh.2026.1980576

**Published:** 2026-10-30

**Authors:** Zhihao Du, Meijuan Zhang, Wenqiong Li, Ziwei Wu

**Affiliations:** 1 School of Physical Education, China University of Mining and Technology, Xuzhou, Jiangsu, China; 2 Beijing Union University, Beijing, China; 3 School of Physical Education, Shandong University of Aeronautics, Binzhou, Shandong, China

**Keywords:** machine learning, model calibration, physical activity, psychological distress, restricted cubic splines, SHAP, sleep quality, university students

## Abstract

**Background:**

Physical activity and sleep disturbance may have nonlinear, interrelated associations with psychological distress. Objective: To develop an internally validated prediction model and explore MVPA–distress nonlinearity and statistical interaction with sleep disturbance.

**Methods:**

A cross-sectional survey included 1,980 undergraduates from 68 classes at one university in Jiangsu Province, China. High-risk distress was defined as K10 ≥ 22. Development and internal testing used 48 classes (*n* = 1,386) and 20 held-out classes (*n* = 594), respectively; tuning used 10-fold GroupKFold. XGBoost was compared with logistic regression, support vector machine, and random forest. Test performance uncertainty was estimated using 2,000 class-level bootstrap resamples. SHAP patterns were examined alongside *post hoc* restricted cubic spline (RCS), interaction, and continuous-K10 analyses.

**Results:**

XGBoost had a test ROC-AUC of 0.838 (95% CI 0.812–0.861), average precision of 0.692 (0.614–0.765), and Brier score of 0.124 (0.108–0.141). At a development-derived threshold of 0.28, sensitivity was 0.739, specificity 0.846, and positive-class F1 0.658. Calibration slope was 1.03 (0.81–1.28), and intercept was −0.04 (−0.22 to 0.15). Cluster-robust RCS testing suggested nonlinearity (*p* = 0.007), with greater uncertainty above approximately 500 min/week. The exploratory MVPA × JSS-4 interaction test yielded *p* = 0.024.

**Conclusion:**

In this single-university sample, XGBoost had the highest observed discrimination among the evaluated models. Nonlinear MVPA associations and statistical interaction with sleep disturbance warrant independent investigation. The results do not establish causal effects, an optimal activity dose, clinical utility, or generalizability to other universities.

## 1 Introduction

Psychological distress is an important public-health concern in university populations. The transition into emerging adulthood coincides with academic pressure, changing social roles, financial concerns, and uncertainty about future employment, while many students also experience disrupted sleep and irregular health behaviors (1, 2). Identifying modifiable behavioral correlates is therefore relevant to campus mental-health surveillance and prevention, while recognizing that observational associations do not by themselves establish causal effects.

Physical activity (PA) is consistently associated with better mental-health outcomes in prospective and meta-analytic research (3, 4). The World Health Organization recommends 150–300 min of moderate-intensity aerobic activity, 75–150 min of vigorous-intensity activity, or an equivalent combination each week for adults (5). These recommendations address general health and should not be treated as mental-health-specific dose thresholds. Moreover, population studies suggest that exercise-mental-health relationships may deviate from a simple linear “more is better” pattern (6, 7).

Many student studies use linear regression terms or coarse PA categories. A single linear term can miss a plateau or other departures from linearity. Flexible prediction models can help generate hypotheses about functional form, but their explanations must be distinguished from causal effects. Regression analyses selected after examining machine-learning results in the same dataset can describe consistency across methods; they do not provide independent confirmation.

Lifestyle behaviors also occur together rather than in isolation. The 24-Hour Movement Guidelines conceptualize physical activity, sedentary behavior, and sleep as interrelated components of daily behavior (8). Studies involving investigators from Karamanoğlu Mehmetbey University have reported relationships among physical activity, smartphone use, sleep quality, and academic functioning in university students (9), and have examined physical activity in students from the Gendarmerie Coast Guard Academy (10). Related research has documented poor sleep and musculoskeletal complaints among university students during the pandemic period (11), while a recent sport-science study with a Karamanoğlu Mehmetbey University co-author found that poorer sleep was associated with higher burnout in elite athletes (12). Military-academy data also demonstrate chronic sleep restriction under highly structured educational demands (13). Together, these studies support examining movement and sleep jointly, but they do not establish a non-linear MVPA-distress dose–response relationship or causal moderation by sleep.

Extreme Gradient Boosting (XGBoost) can capture non-linear functions and high-order interactions without requiring all functional forms to be prespecified (14). SHapley Additive exPlanations (SHAP) can then describe how observed feature values contribute to the fitted model’s predictions (15). However, SHAP values describe model behavior rather than causal treatment effects. Dependence plots may also be influenced by predictor correlation, model specification, and sparse exposure tails. Accordingly, machine-learning patterns are more credible when accompanied by formal regression-based checks, calibration assessment, and careful handling of clustered sampling.

This study aimed to develop and internally validate a class-aware XGBoost classifier for high-risk psychological distress and compare its discrimination and calibration with alternative classifiers. Additional objectives were to characterize MVPA prediction patterns and explore their consistency with conventional regression, assess statistical interaction between MVPA and sleep disturbance, and examine continuous K10 scores. The RCS, GAM, and regression interaction analyses were added during peer review and are treated as *post hoc*, hypothesis-generating analyses rather than independent confirmation of SHAP-derived patterns.

## 2 Materials and methods

### 2.1 Study design and sampling frame

A cross-sectional survey was conducted from September 15 to November 20, 2025, at China University of Mining and Technology in Jiangsu Province, eastern China. The institutional sampling frame comprised 340 eligible full-time undergraduate administrative classes. Sampling was stratified by academic grade (Grades 1–4) and disciplinary division (Engineering and Technology, Natural and Physical Sciences, Humanities and Social Sciences, and Medical/Life Sciences). Sixty-eight classes were selected using a computer-generated pseudorandom sequence (seed 20,250,825). All registered students in the selected classes were invited to participate.

A total of 2,150 questionnaire invitations were distributed through Wenjuanxing (Changsha Ranxing Information Technology Co., Ltd.), and 2,150 completed submissions were recorded; the number of valid questionnaires requires verification. All items required completion before submission. Quality-control exclusions were applied sequentially: 108 submissions failed either of two embedded attention checks, 42 were completed in less than 200 s, and 20 contained implausible IPAQ-SF activity reports. The 200-s threshold was established during pilot testing. These exclusions preceded the class-based split and affected individuals; all 68 classes remained in the analytic sample of 1,980 students. Retained class sizes ranged from 24 to 34 (mean 29.12, SD 2.45). Mandatory item completion prevented item-level missingness in submitted forms but did not ensure that all invited students responded or that all responses were valid. Systematic straightlining screening was not performed. Figure S1 summarizes participant flow.

Participants were eligible if they were full-time undergraduates aged 18–25 years in the sampled classes, could understand the Chinese-language questionnaire, and provided digital informed consent. Exclusion criteria were a reported diagnosis of a severe psychotic disorder, bipolar disorder, or an acute suicidal crisis requiring immediate psychiatric care, or a certified musculoskeletal, neurological, or cardiopulmonary condition that precluded normal physical activity. The protocol was reviewed and approved by the Ethics Committee of the School of Physical Education, China University of Mining and Technology (Approval No. ZZUIRB2025-02). Ethical approval was granted on August 25, 2025, before recruitment and data collection. All participants provided digital informed consent before beginning the survey.

### 2.2 Psychological distress

Psychological distress was assessed using the Chinese version of the 10-item Kessler Psychological Distress Scale (K10), which measures non-specific distress symptoms during the previous 30 days (16, 17). Each item is scored from 1 (“none of the time”) to 5 (“all of the time”), giving a total score of 10–50, with higher scores indicating greater distress. In the present sample, internal consistency was excellent (Cronbach’s α = 0.932; McDonald’s ω = 0.934).

For primary classification, K10 ≥ 22 defined a high-risk screening category combining the high (22–29) and very high (30–50) distress bands. This is an epidemiological screening classification, not a psychiatric diagnosis. Because dichotomization discards information and cut-points may perform differently across settings, continuous K10 scores were examined in *post hoc* sensitivity analyses. These analyses do not validate the threshold for clinical use in this university population.

### 2.3 Physical activity

Physical activity was assessed using the International Physical Activity Questionnaire-Short Form (IPAQ-SF) (18). Participants reported the number of days and average daily duration of vigorous-intensity activity, moderate-intensity activity, and walking during the previous seven days. The primary exposure was weekly moderate-to-vigorous physical activity (MVPA), calculated as moderate minutes/week plus vigorous minutes/week; walking was excluded from the primary MVPA exposure and was described separately.

Data cleaning followed the standard processing guidance of the International Physical Activity Questionnaire (IPAQ) Research Committee (19). Reported durations were converted to minutes/day. Before category-specific truncation, raw reports were screened for physiologically implausible total activity: if the cumulative daily duration of walking, moderate-intensity activity, and vigorous-intensity activity exceeded 960 min/day (>16 h/day), the record was excluded (*n* = 20). For the remaining records, daily durations exceeding 180 min/day in any single activity category (vigorous, moderate, or walking) were truncated to 180 min/day to limit undue leverage from extreme self-reports. Weekly MVPA was then operationalized as MVPA (min/week) = (vigorous days/week × vigorous min/day) + (moderate days/week × moderate min/day). Walking was excluded from the primary MVPA metric and retained as a separate descriptive variable.

### 2.4 Sleep quality

Sleep disturbance was assessed with the four-item Jenkins Sleep Scale (JSS-4) (20). Items assess difficulty falling asleep, waking during the night, difficulty staying asleep, and waking tired or unrefreshed during the previous 30 days. Each item is scored from 0 to 5 according to symptom frequency, yielding a total score of 0–20; higher scores indicate more frequent sleep disturbance. In the current sample, Cronbach’s α was 0.841 and McDonald’s ω was 0.844.

### 2.5 Sociodemographic and lifestyle covariates

Covariates were selected because of their potential relevance to psychological distress and the prediction task. They included age, gender, grade level, academic major, monthly living allowance (RMB), body mass index (BMI; calculated from self-reported height and weight), smoking status, alcohol drinking status, average daily recreational screen time, and academic stress. Academic stress was measured with a single-item 1–10 numeric rating scale from minimal/no stress to extreme/unmanageable stress. The pragmatic single-item assessment is acknowledged as a measurement limitation because it cannot represent the multidimensional nature of academic stress.

### 2.6 Class-aware data partitioning and cross-validation

The 68 classes were partitioned at the cluster level using GroupShuffleSplit (scikit-learn 1.3.2, random_state = 42, n_splits = 1, test_size = 0.294), with Class_ID as the grouping variable. The development set comprised 48 classes (*n* = 1,386), and the class-held-out internal test set comprised 20 classes (n = 594), with zero class overlap. High-risk distress occurred in 322 development participants and 138 test participants (23.23% in each set). Mean class sizes were 28.88 (SD 2.42) and 29.70 (SD 2.47), respectively. As detailed in Supplementary Table S1, the observed distributions of sociodemographic characteristics, behavioral exposures, and psychological distress were broadly comparable between the development and test cohorts. However, the absence of statistically significant differences does not formally demonstrate distributional equivalence or balance. Furthermore, the split was not prospectively stratified by class-level outcome prevalence, and reliance on a single cluster-held-out split introduces potential split instability, which represents an acknowledged study limitation. Hyperparameter selection used 10-fold GroupKFold within development data, holding out intact classes in each validation fold.

### 2.7 Preprocessing, class imbalance, and XGBoost optimization

The train/test partition was created before data-dependent preprocessing. Standardization and one-hot encoding were fitted within each training fold and applied to its validation fold without refitting. The corresponding development-fitted transformations were applied to the held-out test set. Imputation was unnecessary because analyzed variables had no missing values. XGBoost used a fixed scale_pos_weight of 3.30 throughout grouped tuning, derived from the development negative-to-positive ratio of 1,064/322 = 3.304 rather than recalculated in each fold. This approach does not adapt the weight to variation in fold-specific prevalence. Grid search optimized mean validation ROC-AUC. Early stopping used 20 rounds on group-held-out validation folds; the test set did not guide stopping. Table 1 reports the search space and selected configuration. Classification thresholds were selected by the maximum Youden index on development cross-validation predictions. No *post hoc* probability-recalibration model was fitted; XGBoost probabilities were obtained through the logistic link. The frozen pipeline was evaluated on the held-out test set.

**Table 1:** XGBoost hyperparameter search space and selected configuration.

Hyperparameter	Description	Search space/candidates	Selected value
learning_rate	Step-size shrinkage	0.01, 0.03, 0.05, 0.10, 0.20	0.05
max_depth	Maximum tree depth	3, 4, 5, 6, 8	4
min_child_weight	Minimum child instance weight	1, 3, 5, 7	3
subsample	Training-instance subsample ratio	0.6, 0.7, 0.8, 0.9, 1.0	0.80
colsample_bytree	Column subsample ratio	0.6, 0.7, 0.8, 1.0	0.80
n_estimators	Maximum boosting iterations	50, 100, 150, 200, 300, 500	150
gamma	Minimum split-loss reduction	0, 0.05, 0.10, 0.20, 0.50	0.10
reg_lambda	L2 regularization	0.1, 0.5, 1.0, 2.0, 5.0	1.00
scale_pos_weight	Positive-class weighting	Fixed prevalence-based calculation	3.30
early_stopping_rounds	Validation stopping criterion	Fixed	20 rounds

All tuning used class-grouped development cross-validation. The positive-class weight was fixed from development prevalence. Early stopping did not use the internal test set.

### 2.8 Benchmark classifiers

Three benchmark classifiers were trained using the same class-aware development/test framework: ridge-regularized multivariable logistic regression, a radial-basis-function support vector machine (SVM), and a random forest with 300 trees. Model-specific tuning was performed within the grouped cross-validation procedure. These comparators were used to determine whether the flexible gradient-boosted model provided materially better discrimination in this dataset.

### 2.9 Model evaluation, calibration, and statistical comparison

All four classifiers were evaluated on the same 594 students in 20 held-out classes. Discrimination was summarized using ROC-AUC and average precision, the latter representing the reported precision–recall summary and interpreted relative to the positive-outcome prevalence of 138/594 = 0.232. Classification metrics used each model’s development-derived threshold, including 0.28 for XGBoost. Calibration was assessed with a decile plot, Brier score, calibration slope, and calibration intercept. For 2,000 bootstrap replicates, 20 intact test classes were sampled with replacement; all students in each sampled class were retained, including repeated copies when a class was sampled more than once. Performance statistics were recomputed from frozen predictions. Paired cluster-bootstrap intervals summarized differences in ROC-AUC. These intervals quantify uncertainty conditional on the fitted models and observed split, rather than variability from repeating the full model-development process. Conventional paired DeLong comparisons (21) were treated as secondary because pairing alone does not account for within-class dependence. Holm adjustment was applied across the three DeLong comparisons. Cluster-bootstrap differences, rather than conventional DeLong *p* values alone, informed cluster-aware comparisons. No clinical net-benefit analysis was performed.

### 2.10 SHAP interpretability analysis

TreeSHAP was used to explain the optimized XGBoost model (15). Global importance was summarized by mean absolute SHAP values in the held-out test set. Beeswarm plots described the direction and distribution of feature contributions. Dependence plots were used to explore the fitted relationship between weekly MVPA and model output, and SHAP interaction values were used to visualize pairwise structure involving MVPA, sleep quality, and screen time. Rug plots and marginal density information were included to show observation density across MVPA and to make sparse upper-tail patterns visible. SHAP values were interpreted only as contributions to model predictions; they were not treated as causal effects, clinical thresholds, or estimates of individual treatment benefit.

### 2.11 Restricted cubic spline regression

A multivariable logistic RCS model was fitted for binary high-risk distress (22). Four knots were placed at the 5th, 35th, 65th, and 95th MVPA percentiles (20, 150, 240, and 450 min/week). Adjustment included age, gender, grade, academic major, monthly allowance, BMI, smoking, alcohol use, screen time, academic stress, and JSS-4. Huber–White standard errors clustered by Class_ID were used for Wald tests of the overall association and joint nonlinear terms. Odds ratios were expressed relative to 150 min/week, a reference within the observed activity distribution, rather than an estimated optimum. The unweighted sum of moderate and vigorous activity minutes is not equivalent to guideline-equivalent moderate activity minutes. Spline estimates were considered alongside the observed exposure density. This *post hoc* analysis was selected after inspection of model patterns and does not constitute independent confirmation.

### 2.12 Formal statistical interaction testing

A separate multivariable logistic regression evaluated the statistical interaction between physical activity and sleep disturbance using mean-centered continuous terms:

$$A=\text{MVPA}/100-1.94;S=\mathrm{JS}S_{4}-5.60$$

$$\text{logit}(p)=\beta_{0}+\beta ₁A+\beta ₂S+\beta ₃\mathrm{AS}+\gamma^{\prime }Z$$

Here, p is the predicted probability of high-risk distress. A denotes weekly MVPA divided by 100 min/week and centered using 1.94, and S denotes JSS-4 centered using 5.60. These rounded centering constants correspond approximately to the sample means. Mean-centering was used to reduce nonessential collinearity between the main terms and their product; reported variance inflation factors were below 2.1. In this parameterization, β₁ describes the MVPA association at JSS-4 = 5.60, β₂ describes the JSS-4 association at MVPA = 194 min/week, and β₃ describes interaction on the log-odds scale. The adjustment variables Z were age, gender, grade, academic major, monthly allowance, BMI, smoking, alcohol use, screen time, and academic stress. Huber–White standard errors were clustered by Class_ID.

To translate the model into an interpretable absolute risk scale, model-adjusted marginal predicted probabilities of psychological distress across the MVPA continuum (0 to 600 min/week) were computed at representative sleep disturbance scores of JSS-4 = 2, 6, and 10 (evaluated with all other covariates averaged over their observed sample distribution), accompanied by cluster-robust 95% confidence intervals derived via the delta method. These representative scores illustrate the fitted model across the observed range of sleep disturbance rather than defining validated clinical cutoff categories. This analysis evaluates statistical interaction in observational data, not causal effect modification.

### 2.13 Continuous-outcome sensitivity analysis

To assess whether the binary K10 threshold created an artificial functional pattern, two continuous-outcome analyses were performed. First, an XGBoost regressor predicted continuous K10 total score (10–50). Second, a generalized additive model (GAM) used a penalized thin-plate spline for MVPA while adjusting for the same covariates. The estimated degrees of freedom and significance of the smooth term were used to assess whether the non-linear pattern persisted without outcome dichotomization. These analyses were exploratory and used the same dataset as the primary analyses; agreement across models does not constitute independent replication.

### 2.14 Software and reproducibility

Machine-learning analyses were performed in Python 3.10.12 using xgboost 2.0.3, SHAP 0.44.1, and scikit-learn 1.3.2. RCS, calibration, DeLong tests, and GAM analyses were performed in R 4.3.2 using the rms, pROC, and mgcv packages. Two-sided *p* values were interpreted as exploratory evidence. Holm adjustment was limited to the three secondary DeLong comparisons; the spline and interaction tests were not treated as confirmatory. Reporting was revised with reference to STROBE principles for cross-sectional observational studies.

## 3 Results

### 3.1 Participant characteristics and missing data

The final sample comprised 1,980 students from 68 classes. Mean age was 20.4 years (SD 1.6; range 18–25), and 1,079 participants (54.5%) were female. Because the online questionnaire required completion of all items before submission, the analyzed dataset contained 0.0% missing values for every variable included in Table 2. A total of 460 participants (23.2%) met the high-risk K10 threshold of ≥22.

**Table 2:** Participant characteristics and variable distributions (*N* = 1,980).

Variable	Category/unit	Total (*N* = 1,980)	High distress (*n* = 460)	Low distress (*n* = 1,520)	Missing %
Age	years, mean ± SD	20.4 ± 1.6	20.5 ± 1.7	20.3 ± 1.5	0.0
Gender	Male, *n* (%)	901 (45.5%)	185 (40.2%)	716 (47.1%)	0.0
	Female, *n* (%)	1,079 (54.5%)	275 (59.8%)	804 (52.9%)	0.0
Grade	Freshman, *n* (%)	524 (26.5%)	108 (23.5%)	416 (27.4%)	0.0
	Sophomore, *n* (%)	510 (25.8%)	122 (26.5%)	388 (25.5%)	0.0
	Junior, *n* (%)	486 (24.5%)	120 (26.1%)	366 (24.1%)	0.0
	Senior, *n* (%)	460 (23.2%)	110 (23.9%)	350 (23.0%)	0.0
Academic major	Engineering, *n* (%)	752 (38.0%)	170 (37.0%)	582 (38.3%)	0.0
	Science, *n* (%)	475 (24.0%)	115 (25.0%)	360 (23.7%)	0.0
	Humanities/Arts, *n* (%)	495 (25.0%)	118 (25.7%)	377 (24.8%)	0.0
	Medical/Life, n (%)	258 (13.0%)	57 (12.4%)	201 (13.2%)	0.0
Monthly allowance	RMB, mean ± SD	2,150 ± 620	2,110 ± 645	2,162 ± 612	0.0
MVPA	min/week, mean ± SD	193.7 ± 131.2	112.5 ± 88.4	218.3 ± 132.1	0.0
Walking	min/week, mean±SD	245.2 ± 142.6	238.1 ± 140.2	247.4 ± 143.3	0.0
Sleep disturbance (JSS-4)	score 0–20, mean ± SD	5.6 ± 3.1	8.8 ± 3.6	4.6 ± 2.2	0.0
Screen time	hours/day, mean±SD	6.3 ± 2.2	7.7 ± 2.5	5.9 ± 1.9	0.0
BMI	kg/m^2^, mean ± SD	21.4 ± 3.2	21.6 ± 3.5	21.3 ± 3.0	0.0
Smoking	Current smoker, *n* (%)	168 (8.5%)	49 (10.7%)	119 (7.8%)	0.0
Alcohol drinking	Drinker, *n* (%)	342 (17.3%)	88 (19.1%)	254 (16.7%)	0.0
Academic stress	score 1–10, mean ± SD	6.0 ± 1.9	7.8 ± 1.6	5.5 ± 1.7	0.0
K10 total score	score 10–50, mean ± SD	17.8 ± 6.0	26.6 ± 4.3	15.2 ± 3.4	0.0

Values are means ± SD or counts (percentages). High distress was defined as K10 ≥ 22. All analyzed variables had no missing submitted items. These descriptive comparisons do not establish causality.

The high-distress group reported lower weekly MVPA (112.5 ± 88.4 vs. 218.3 ± 132.1 min/week), greater sleep disturbance (JSS-4 8.8 ± 3.6 vs. 4.6 ± 2.2), longer screen time (7.7 ± 2.5 vs. 5.9 ± 1.9 h/day), and higher academic stress (7.8 ± 1.6 vs. 5.5 ± 1.7). These unadjusted descriptive differences do not establish temporal or causal relationships.

### 3.2 Predictive performance, calibration, and model comparison

On the class-held-out internal test set, XGBoost had ROC-AUC 0.838 (95% CI 0.812–0.861) and average precision 0.692 (0.614–0.765). At the development-derived threshold of 0.28, accuracy was 0.822, sensitivity 0.739, specificity 0.846, PPV 0.593, NPV 0.915, and positive-class F1 0.658. The Brier score was 0.124 (0.108–0.141), calibration slope 1.03 (0.81–1.28), and calibration intercept −0.04 (−0.22 to 0.15). These calibration estimates suggest approximate agreement within this split, with uncertainty that remains relevant for only 20 test classes. Table 3 reports model performance and intervals.

**Table 3:** Model performance in 594 participants from 20 held-out classes.

(A) Shows discrimination and calibration				
Model	ROC-AUC (95% CI)	Average precision (95% CI)	Brier score (95% CI)	Calibration slope (95% CI)
Logistic regression	0.756 (0.724–0.787)	0.541 (0.462–0.623)	0.168 (0.150–0.187)	0.86 (0.68–1.06)
SVM (RBF)	0.779 (0.748–0.809)	0.584 (0.505–0.661)	0.152 (0.134–0.171)	0.91 (0.72–1.12)
Random forest	0.804 (0.776–0.832)	0.638 (0.561–0.714)	0.139 (0.122–0.157)	0.97 (0.76–1.20)
XGBoost	0.838 (0.812–0.861)	0.692 (0.614–0.765)	0.124 (0.108–0.141)	1.03 (0.81–1.28)

(B) Threshold-dependent performance with 95% confidence intervals.				
Metric	Logistic regression	SVM (RBF)	Random forest	XGBoost
Threshold	0.24	0.26	0.27	0.28
Accuracy	0.737 (0.697–0.774)	0.764 (0.726–0.801)	0.788 (0.751–0.823)	0.822 (0.785–0.854)
Sensitivity	0.616 (0.528–0.701)	0.645 (0.561–0.727)	0.703 (0.621–0.783)	0.739 (0.655–0.818)
Specificity	0.774 (0.731–0.815)	0.800 (0.758–0.841)	0.814 (0.773–0.852)	0.846 (0.804–0.885)
Positive-class F1	0.521 (0.455–0.587)	0.560 (0.493–0.624)	0.606 (0.542–0.669)	0.658 (0.602–0.718)
PPV	0.452 (0.380–0.526)	0.494 (0.420–0.571)	0.533 (0.457–0.609)	0.593 (0.518–0.672)
NPV	0.869 (0.832–0.903)	0.882 (0.847–0.914)	0.900 (0.867–0.931)	0.915 (0.884–0.943)

(C) Pairwise ROC-AUC differences and secondary DeLong comparisons.			
XGBoost versus	ΔROC-AUC (cluster 95% CI)	DeLong P	Holm-adjusted P
Logistic regression	0.082 (0.048–0.117)	<0.001	<0.001
SVM	0.059 (0.027–0.092)	<0.001	<0.001
Random forest	0.034 (0.004–0.065)	0.018	0.018

(A) XGBoost calibration intercept: −0.04 (95% CI −0.22 to 0.15). Average precision is interpreted relative to the test prevalence of 0.232. (B) PPV, positive predictive value; NPV, negative predictive value. F1 refers to the positive class. Thresholds were selected on development cross-validation predictions. (C) Intervals use 2,000 class-level bootstrap replicates. Holm adjustment applies to three DeLong tests; conventional DeLong tests do not account for class clustering. The paired cluster-bootstrap intervals provide the class-aware comparison.

The observed ROC-AUC differences favoring XGBoost were 0.082 versus logistic regression (paired cluster-bootstrap 95% CI 0.048–0.117), 0.059 versus SVM (0.027–0.092), and 0.034 versus random forest (0.004–0.065). Secondary paired DeLong P values were <0.001, <0.001, and 0.018; Holm-adjusted values were <0.001, <0.001, and 0.018, respectively. These findings concern comparative discrimination in this internal dataset, not external validity or clinical utility.

### 3.3 Global SHAP feature importance

Global SHAP analysis ranked sleep disturbance (JSS-4) as the most influential feature in the fitted XGBoost model, followed by weekly MVPA, screen time, academic stress, and age. Higher JSS-4 scores tended to have positive SHAP values, whereas higher MVPA values tended to have negative SHAP values over much of the observed range. These rankings describe predictive importance within the fitted model rather than etiological importance. Figure 1 shows the global ranking and the distribution of feature contributions.

**Figure 1:**
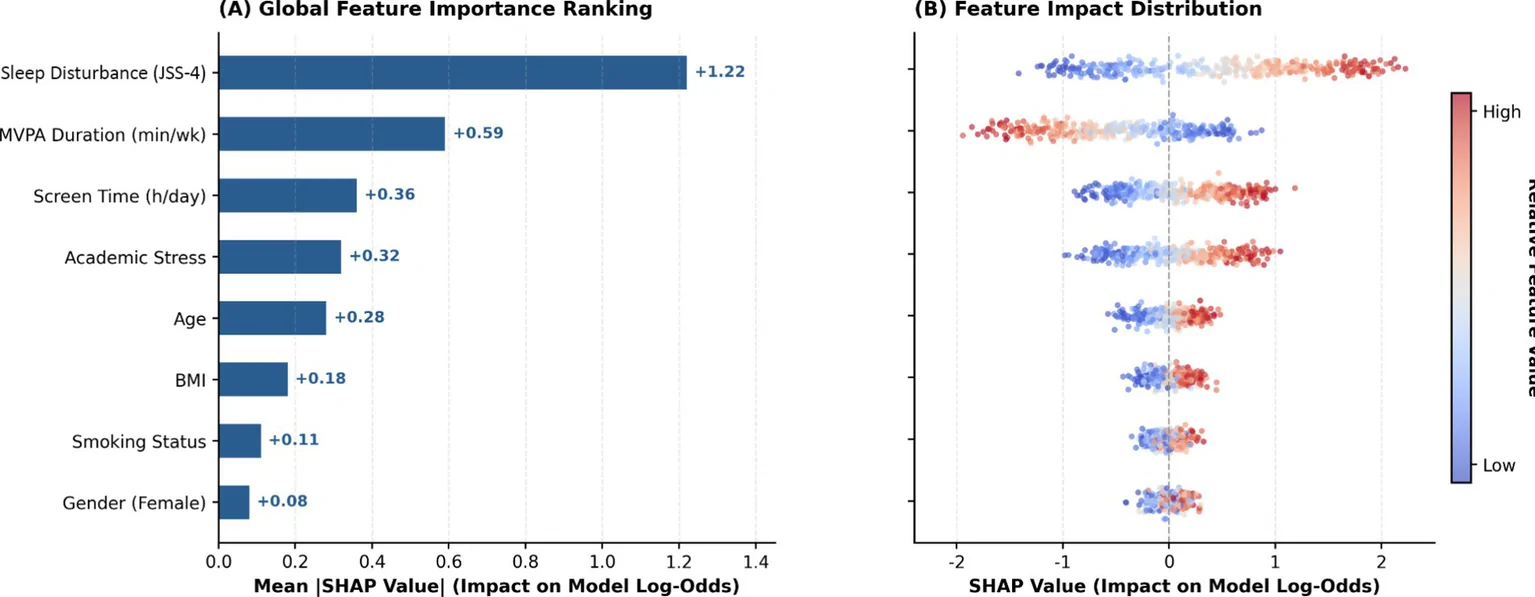
Global SHAP analysis of the XGBoost model. **(A)** Feature importance ranked by mean absolute SHAP value in the class-held-out test set. **(B)** Beeswarm distribution showing the direction and dispersion of feature contributions to model output.

### 3.4 Non-linear MVPA pattern and RCS robustness analysis

The SHAP dependence plot (Figure 2A) showed a non-linear, non-monotonic prediction pattern across weekly MVPA. SHAP values declined sharply from near-zero activity toward approximately 200 min/week, remained generally lower across roughly 200–400 min/week, and became more dispersed above approximately 500 min/week. The rug and density information show that observations were relatively sparse in this upper tail; the apparent upward tendency at very high MVPA should therefore be interpreted as uncertain model behavior rather than evidence that high activity causes psychological harm.

**Figure 2:**
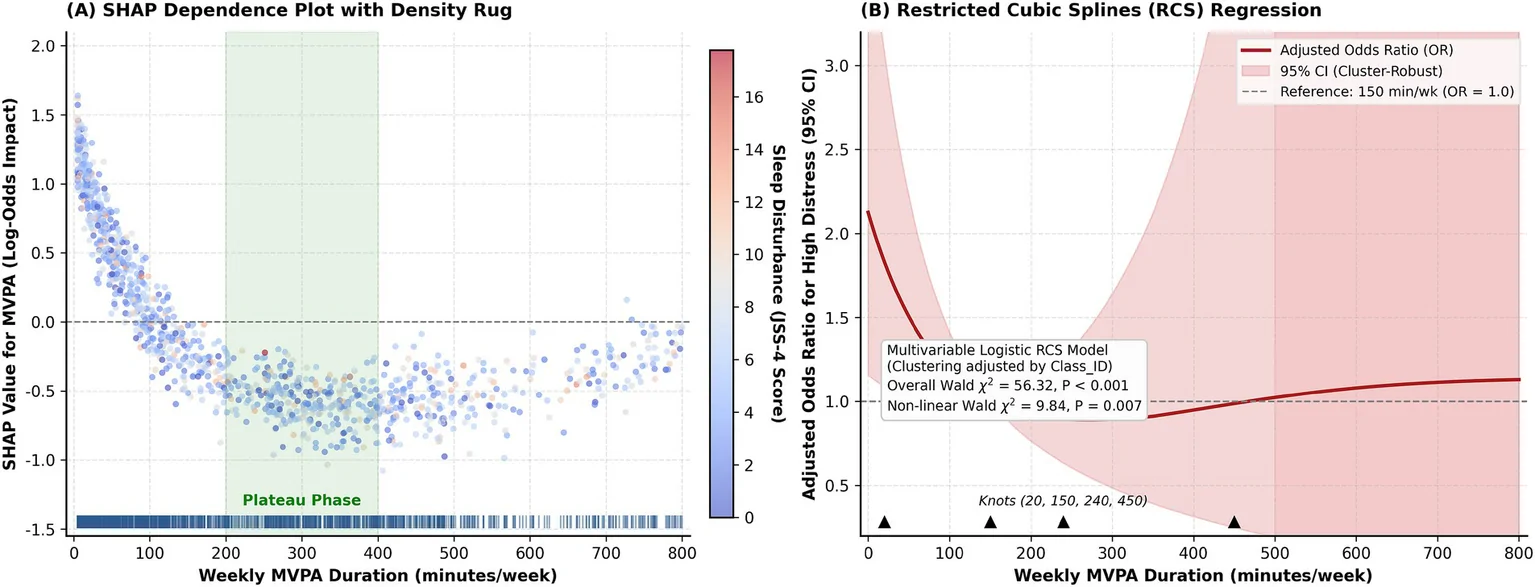
Exploratory nonlinear modeling of weekly MVPA and psychological distress. **(A)** SHAP dependence with observation density. **(B)** Covariate-adjusted logistic RCS odds ratios relative to 150 min/week, with class-cluster-robust 95% confidence bands and knots at 20, 150, 240, and 450 min/week. Overall Wald χ^2^ = 56.32, *p* < 0.001; nonlinear Wald χ^2^ = 9.84, *p* = 0.007. Sparse observations above approximately 500 min/week limit upper-tail interpretation.

The *post hoc* class-cluster-robust RCS analysis (Figure 2B) suggested nonlinearity (nonlinear Wald χ^2^ = 9.84, *p* = 0.007; overall Wald χ^2^ = 56.32, *p* < 0.001). Relative to 150 min/week, adjusted odds were higher at 0 min/week (OR 2.14, 95% CI 1.61–2.84) and lower at 250 min/week (OR 0.81, 0.68–0.96). Estimates were imprecise at 600 min/week (OR 0.79, 0.49–1.28) and 800 min/week (OR 0.91, 0.44–1.88). These exploratory results describe consistency across methods in the same sample; they do not identify an optimal dose or a validated threshold.

### 3.5 Statistical interaction between MVPA and sleep quality

In the mean-centered multivariable logistic regression model, both main terms were statistically significant: MVPA100 centered (β1 = −0.184, SE = 0.042, Wald χ2 = 19.18, *p* < 0.001) and JSS-4 centered (β2 = 0.286, SE = 0.025, Wald χ2 = 130.87, *p* < 0.001). The multiplicative interaction term was positive and statistically significant (β3 = 0.041 per 100 min/week × JSS-4 point; cluster-robust SE = 0.018; Wald χ2 = 5.12; *p* = 0.024; interaction OR = 1.042, 95% CI 1.005–1.080). The positive interaction coefficient indicates that the inverse association between physical activity and distress becomes attenuated as sleep disturbance increases.

Figure 3A shows the SHAP interaction scatter visualization for weekly MVPA and sleep disturbance across descriptive JSS-4 groups. Figure 3B depicts model-adjusted marginal predicted probabilities across MVPA volumes at representative sleep disturbance scores (JSS-4 = 2, 6, and 10). For students reporting low sleep disturbance (JSS-4 = 2), physical activity exhibited a marked inverse association: the predicted distress probability declined sharply from 18.2% (95% CI 14.8–21.6%) at 0 min/week to 7.9% (95% CI 5.8–10.0%) at 300 min/week. For students at the sample mean sleep score (JSS-4 = 6), the probability decreased from 31.4% (95% CI 27.2–35.6%) to 23.6% (95% CI 20.2–27.0%) at 300 min/week. In contrast, for students experiencing severe sleep disturbance (JSS-4 = 10), the absolute probability of distress remained chronically elevated across the entire activity spectrum (48.6% [95% CI 42.8–54.4%] at 0 min/week and 48.1% [95% CI 42.1–54.1%] at 300 min/week), illustrating that severe sleep disturbance substantially attenuates the predictive protective association of physical activity. This pattern demonstrates observational behavioral heterogeneity on the probability scale rather than causal effect modification.

**Figure 3:**
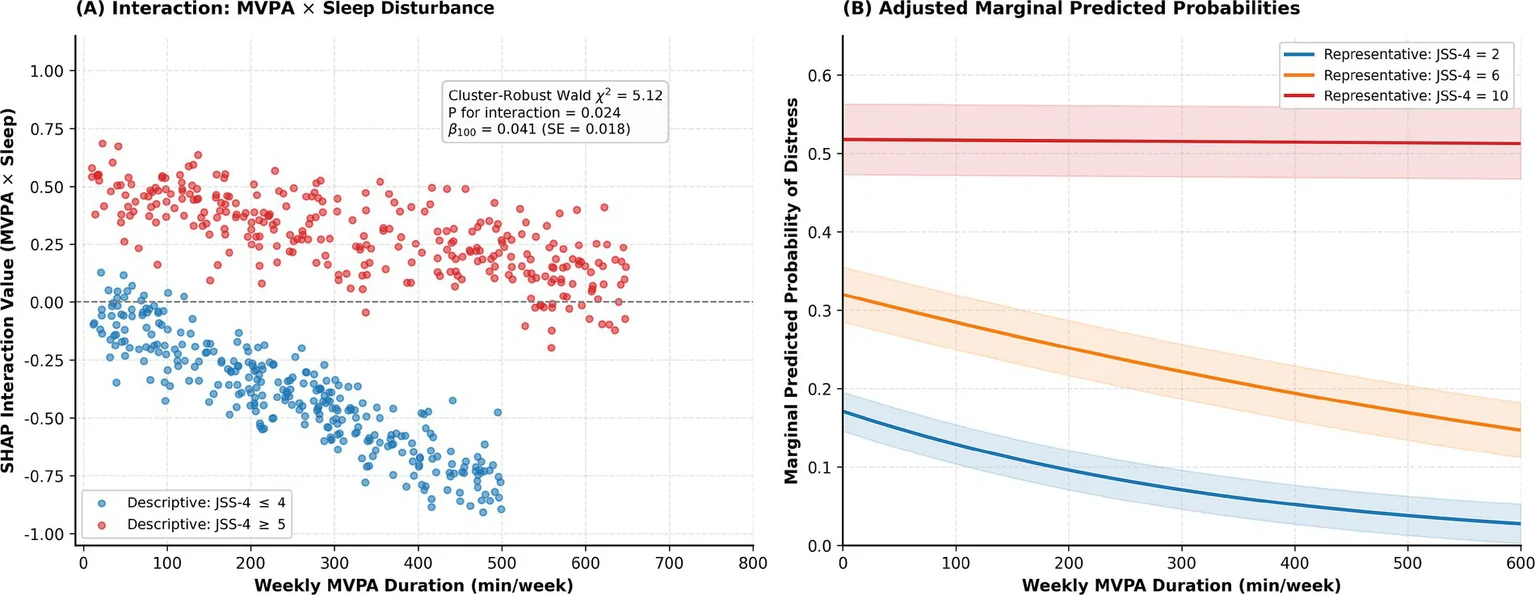
Exploratory MVPA–sleep statistical interaction. **(A)** SHAP interaction scatter visualization across descriptive sleep groups (JSS-4 ≤ 4 vs. ≥ 5). **(B)** Adjusted marginal predicted distress probabilities across the weekly MVPA continuum stratified by representative sleep disturbance scores of JSS-4 = 2, 6, and 10, with cluster-robust 95% confidence bands. The text box reports the post-hoc multivariable logistic product-term test (β = 0.041 per 100 min/week × JSS-4 point, cluster-robust SE = 0.018, Wald χ2 = 5.12, *p* = 0.024). These curves depict observational predictions on an absolute probability scale and do not establish causal effect modification.

### 3.6 Individual-level model explanations

Individual SHAP visualizations illustrate how different combinations of observed characteristics contribute to model predictions. Such decompositions describe the fitted model; they do not demonstrate that changing one feature would change an individual student’s psychological outcome. Figure 4 provides an overview of contributions across 100 observations.

**Figure 4:**
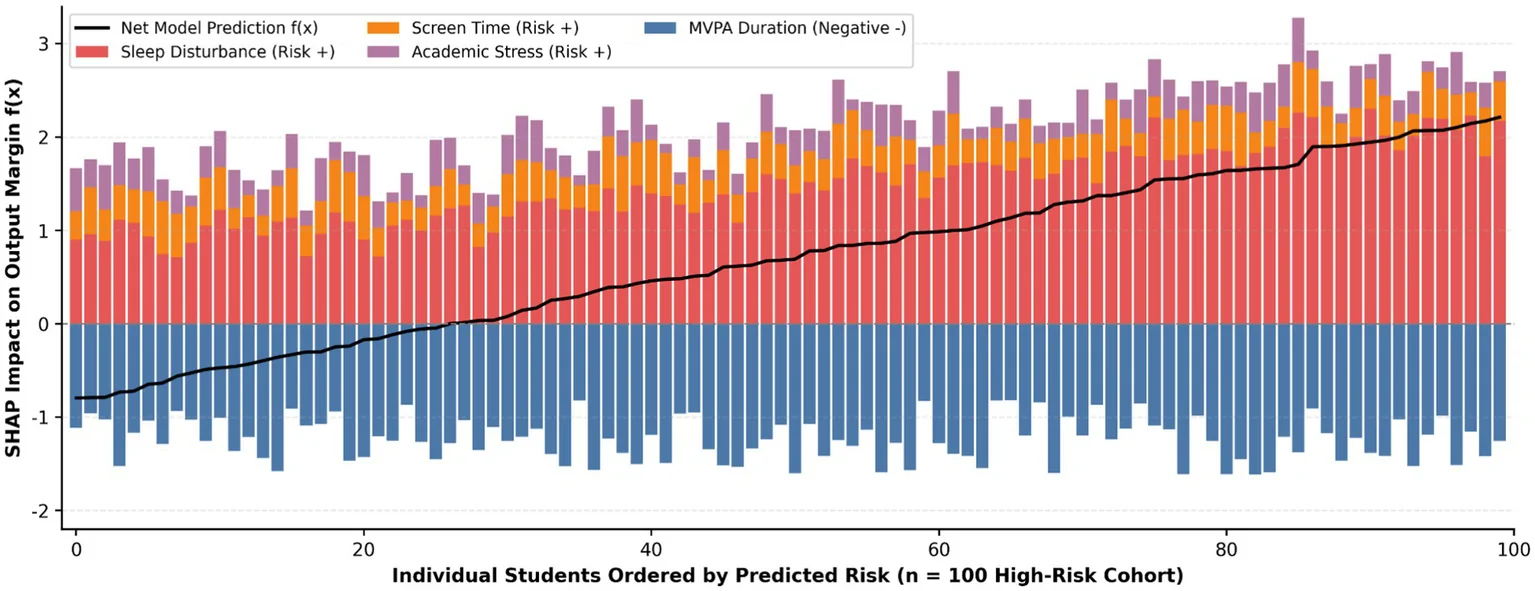
Stacked SHAP force visualization across 100 individuals classified as high-risk distress. Individual predictions are ordered along the horizontal axis by increasing predicted risk. Colored stacked segments illustrate the major feature attributions (sleep disturbance, screen time, academic stress, and MVPA duration). The solid black trajectory denotes the net model margin f(x) , which equals the expected base value E[f(x)]=−0.524 plus the sum of all feature attributions across the full feature space (with minor unplotted covariates aggregated into the net prediction margin).

For illustrative Student #142 (Figure 5), the final prediction margin was −1.44. Applying the logistic link gives a predicted probability of approximately 19.2%, below the 0.28 classification threshold. This transformation is not an empirical probability-calibration procedure. The example is a descriptive explanation of a fitted prediction, not an individualized exercise prescription.

**Figure 5:**
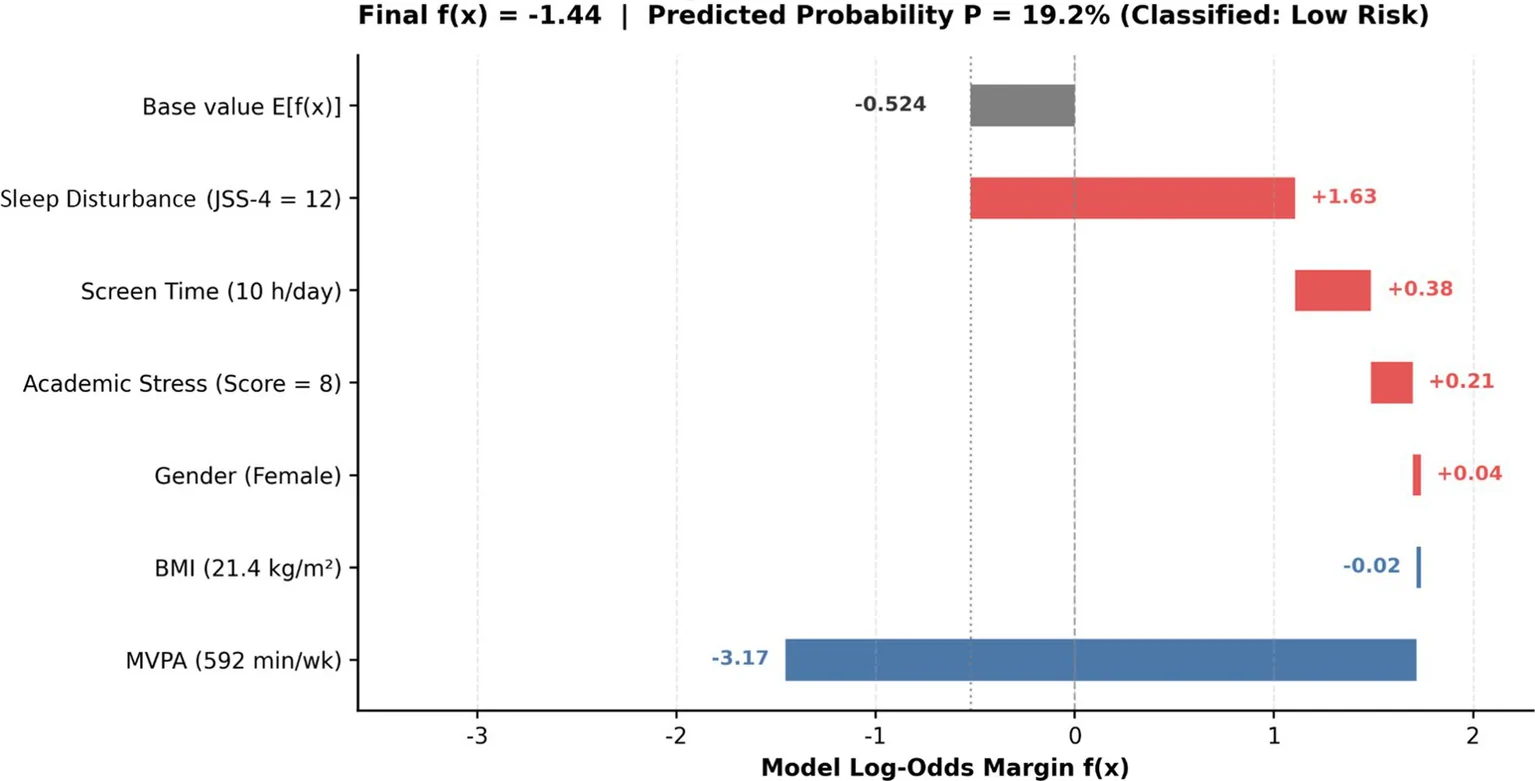
SHAP waterfall decomposition for illustrative Student #142. Feature contributions sum with the model baseline to a final margin of −1.44, corresponding to a predicted probability of approximately 19.2% through the logistic link. No *post hoc* empirical probability recalibration was performed.

### 3.7 Continuous K10 sensitivity analysis

In the original continuous-outcome sensitivity analyses, the XGBoost regressor achieved R^2^ = 0.412 and RMSE = 5.12 on the class-held-out internal test set. Sleep disturbance and MVPA remained prominent predictive features. The adjusted GAM reported a nonlinear MVPA smooth (*F* = 8.42; estimated degrees of freedom = 3.65; nominal *p* < 0.001), with a decline at lower activity levels and a flatter intermediate region. These *post hoc* results suggest that the pattern was not confined to a dichotomized outcome. Because class-adjusted uncertainty for this GAM is not established here, its nominal P value is not used as additional cluster-adjusted confirmation.

Figure 6 summarizes the binary classifier evaluation through ROC, precision–recall, and calibration displays on the class-held-out internal test set.

**Figure 6:**
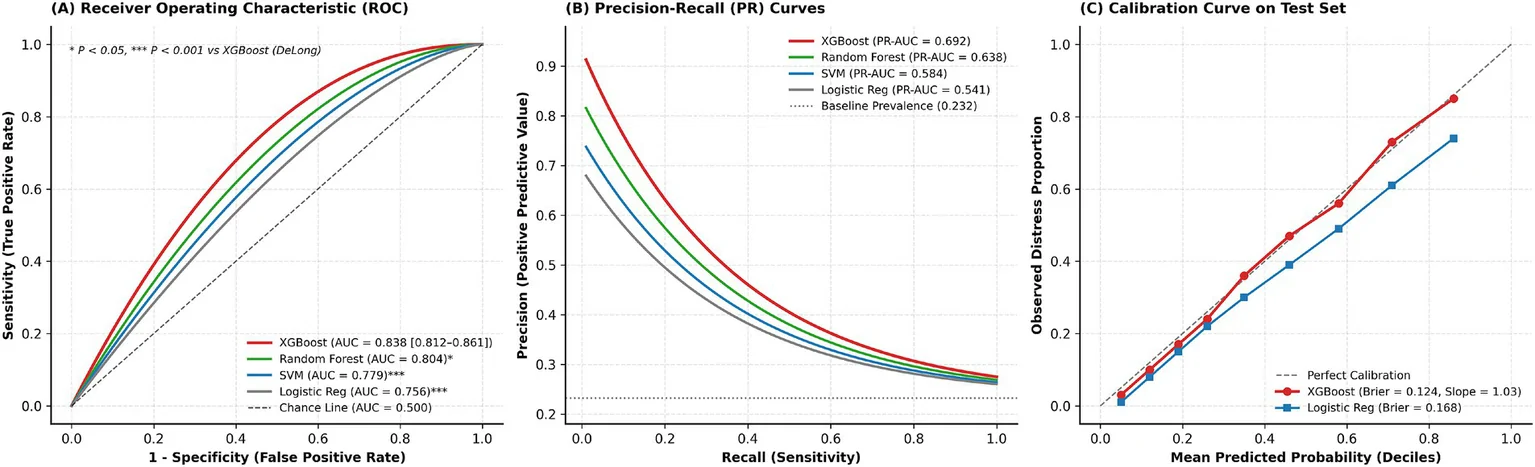
Predictive evaluation on the class-held-out internal test set of 594 students in 20 classes. **(A)** ROC curves; XGBoost ROC-AUC 0.838 (95% CI 0.812–0.861). **(B)** Precision–recall curves with average-precision summaries and a prevalence reference of 0.232. **(C)** Calibration by probability decile; XGBoost Brier score 0.124, calibration slope 1.03, and intercept −0.04. Uncertainty estimates use class-level resampling.

## 4 Discussion

### 4.1 Principal findings

In this cross-sectional sample of 1,980 students from one university, XGBoost showed the highest observed internal discrimination among the evaluated classifiers. Calibration estimates were compatible with approximate agreement between predicted and observed probabilities, although uncertainty remained. SHAP and exploratory RCS analyses described nonlinear MVPA patterns with a sparse upper tail. A *post hoc* regression product term suggested statistical interaction between MVPA and sleep disturbance. Continuous-K10 analyses provided a further descriptive sensitivity check. None of these analyses independently validates a behavioral threshold or establishes clinical utility.

### 4.2 Non-linear MVPA-distress pattern

The principal substantive finding was that the observed relationship between MVPA and psychological distress was not well represented by a single linear term. Both modeling approaches showed the largest change across the low-to-intermediate activity range, followed by a comparatively flatter region. This morphology is broadly compatible with population evidence that exercise-mental-health associations may be non-linear (6, 7). It should not, however, be interpreted as identifying a therapeutic window. The WHO recommendation of 150–300 min/week of moderate-intensity aerobic activity provides a public-health reference point, but the present cross-sectional analysis cannot redefine that recommendation for psychological distress (5).

The upper tail is especially important for interpretation. Participant density declined sharply above approximately 500 min/week, the SHAP distribution widened, and RCS confidence intervals became broad. Consequently, the modest upward tendency in model output at high MVPA may reflect sampling sparsity, model uncertainty, differences in exercise context, reverse causation, or residual confounding. The data do not demonstrate that high physical activity causes distress or that students should avoid activity above any particular level.

### 4.3 Physical activity, sleep quality, and the relevant literature

Sleep disturbance was the most influential XGBoost feature, and the formal MVPA × JSS-4 term indicated statistical interaction. This finding is consistent with a broader behavioral framework in which movement, sedentary time, and sleep co-occur within the same daily time budget (8, 23). It is also compatible with university-student evidence linking physical activity, smartphone use, and sleep quality (9), with pandemic-era findings on student sleep and musculoskeletal symptoms (11), and with research involving Gendarmerie Coast Guard Academy students by investigators affiliated with Karamanoğlu Mehmetbey University (10). Military-academy actigraphy data similarly show that highly structured academic environments can be accompanied by chronic sleep restriction (13). More recent sport-science evidence has also linked poorer sleep with higher burnout in intensively trained athletes (12). University-student research has also examined physical activity in relation to mental health and well-being (24). Reviews discuss potential biological and psychosocial pathways linking activity and depression (25), including BDNF-related mechanisms studied in acute exercise and cognition (26), as well as exercise-program characteristics in depression treatment (27). Research on physical activity and sleep includes a systematic review (28) and a university-student study conducted during an academic stress period (29). These studies provide context rather than validation of the present observational interaction.

The present findings motivate further joint investigation of physical activity and sleep. Their interpretation remains observational: sleep disturbance, activity, academic stress, and psychological distress may influence one another or share unmeasured determinants. In particular, a statistically detectable product term does not establish a practically important probability difference or show that improving sleep would increase the mental-health benefit of exercise. Independent data and prespecified models are required to evaluate reproducibility.

### 4.4 Predictive modeling, calibration, and interpretability boundaries

Separating classes between development and testing reduces the risk that shared class characteristics inflate internal performance estimates. This partitioning does not itself account for dependence in uncertainty estimates. Class-level bootstrap resampling and class-cluster-robust regression standard errors address that separate issue. However, test performance remains conditional on a single allocation of 68 classes, with only 20 classes contributing to test uncertainty. Discrimination, calibration, and clinical utility are distinct: neither ROC-AUC nor a favorable calibration estimate establishes screening net benefit. No decision-curve analysis or other direct net-benefit evaluation was conducted.

SHAP further improved transparency by showing how observed variables contributed to model output. However, a negative SHAP value for MVPA means only that the observed MVPA value shifted the model prediction in a lower-risk direction relative to the model baseline. It does not imply that prescribing more MVPA to that individual would cause an equivalent reduction in distress. The current model should therefore be viewed as a research and hypothesis-generation tool rather than a clinical decision-support system.

### 4.5 Strengths and limitations

Strengths include a defined institutional sampling frame, class-separated development and testing, grouped model tuning, explicit reporting of class weighting, discrimination and calibration assessment, class-aware uncertainty estimation, exploratory spline and interaction analyses, observation-density displays, and continuous-outcome sensitivity analyses.

Several limitations remain. The cross-sectional design prevents determination of temporal or causal direction, and reverse causation is plausible. Participants came from one university in Jiangsu; institutional sampling does not establish representativeness of Chinese university students generally. Validation was internal, and reliance on a single class-held-out split may produce unstable performance rankings across other allocations. Conditional test-set bootstrap intervals do not include the full uncertainty from repeated model training and selection. Self-reported activity, sleep, height, weight, screen time, and other covariates are susceptible to recall and social-desirability error. Mandatory completion prevents missing submitted items but does not ensure response validity; systematic straightlining screening was not performed. Single-item academic stress may leave residual confounding. Other unmeasured determinants include mental-health history, medication, social support, diet, and exercise context. Sparse observations above approximately 500 min/week limit interpretation of the upper tail. The RCS and interaction analyses were selected after examining the same data and are exploratory; their nominal P values do not provide independent confirmation. Dichotomization also discards information, and the continuous-outcome GAM does not provide a separate cluster-adjusted confirmation. Neither external validation nor prospective clinical-utility evaluation has been performed.

### 4.6 Future research

Future work should replicate the observed functional shape in independent universities and geographic regions, preferably using prospective repeated-measure designs. Objective accelerometry and device-based sleep assessment would reduce self-report error. Prespecified RCS or GAM models should be combined with class- or institution-level correlation structures where appropriate, and future studies should test whether the MVPA × sleep interaction replicates across cohorts. If a stable turning region emerges prospectively, change-point models with bootstrap uncertainty could evaluate its reproducibility. Ultimately, randomized or quasi-experimental studies would be required to determine whether changing activity or sleep causes changes in psychological distress and whether combined behavioral interventions offer benefits beyond single-component programs.

## 5 Conclusion

In this single-university cross-sectional sample, XGBoost had the highest observed discrimination among four classifiers evaluated using class-held-out internal testing. SHAP and *post hoc* regression analyses suggested nonlinear MVPA associations and statistical interaction with sleep disturbance. These hypothesis-generating findings do not establish a causal effect, an optimal exercise dose, or clinical screening utility. Independent institutional and geographic validation, prospective follow-up, and prespecified analyses are needed before practical application.

## Data Availability

The raw, de-identified dataset is not publicly available because of institutional ethical restrictions and privacy protections for sensitive student mental-health data. A minimal anonymized dataset supporting the reported findings may be made available by the corresponding author upon reasonable academic request, subject to formal institutional approval and an appropriate data-transfer agreement.
