# Supplementary material for: Nonlinear associations between physical activity, sleep quality, and psychological distress in university students: a cross-sectional interpretable machine learning study
Source: Front Public Health. 2026 Oct 30;14:1980576. doi: 10.3389/fpubh.2026.1980576 (PMC13760571; doi:10.3389/fpubh.2026.1980576)
Supplement: Supplementary file 1 [file data_sheet_1.pdf]

Supplementary material

Nonlinear associations between physical activity, sleep quality, and psychological distress in university students: a cross-sectional interpretable machine learning study

Wenqiong Li[1] and Zhihao Du[2]*

1 School of Physical Education, Shandong University of Aeronautics and Astronautics, Binzhou, Shandong, China
2 School of Physical Education, China University of Mining and Technology, Xuzhou, Jiangsu, China

*Correspondence: Zhihao Du; dzh_cumt_edu@163.com

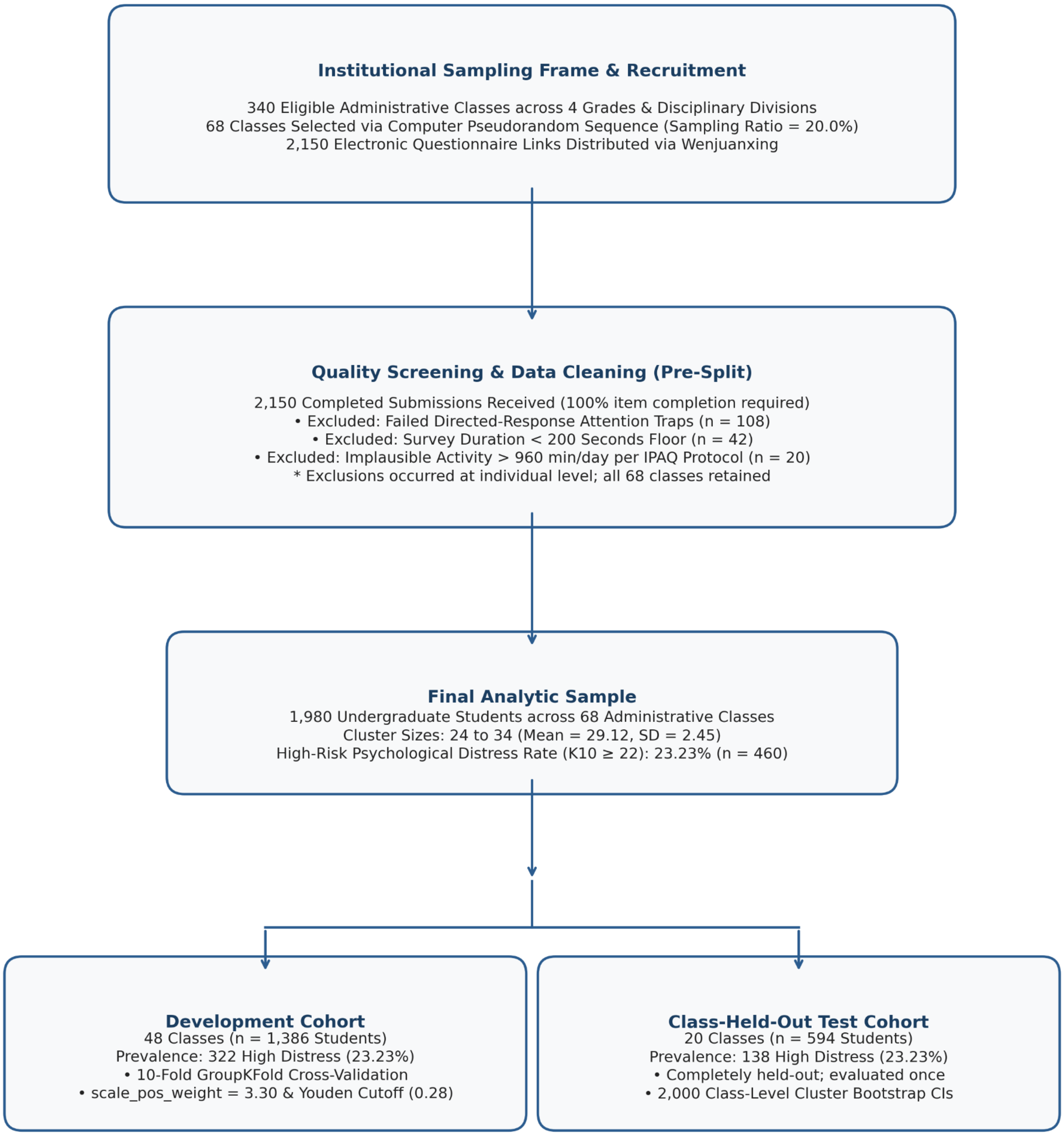

Figure S1. Participant flow and class-based allocation. Of 2,150 completed submissions, 108 failed attention checks, 42 were completed in less than 200 seconds, and 20 had implausible IPAQ-SF values. The 1,980 retained students belonged to 68 classes, allocated to development (48 classes; n=1,386) and internal testing (20 classes; n=594).

Supplementary Table S1. Characteristics of the development and internal test cohorts.

Characteristic	Development<br>48 classes; n=1,386	Internal test<br>20 classes; n=594	Statistic	P value
Cluster Size (Students/Class)	28.88 ± 2.42 (range 24–34)	29.70 ± 2.47 (range 25–34)	t = -1.27	0.207
High Distress (K10 ≥ 22)	322 (23.23%)	138 (23.23%)	χ^2 = 0.000	1.000
Low Distress (K10 < 22)	1,064 (76.77%)	456 (76.77%)	χ^2 = 0.000	1.000
Age (years)	20.4 ± 1.6	20.4 ± 1.5	t = 0.33	0.742
Biological Sex, n (%)			χ^2 = 0.133	0.716
- Male	627 (45.24%)	274 (46.13%)		
- Female	759 (54.76%)	320 (53.87%)		
Academic Grade, n (%)			χ^2 = 0.083	0.994
- Freshman	365 (26.33%)	159 (26.77%)		
- Sophomore	359 (25.90%)	151 (25.42%)		
- Junior	341 (24.60%)	145 (24.41%)		
- Senior	321 (23.16%)	139 (23.39%)		
Disciplinary Division, n (%)			χ^2 = 0.104	0.991
- Engineering & Technology	525 (37.88%)	227 (38.22%)		
- Natural Sciences	334 (24.10%)	141 (23.74%)		
- Humanities & Social Sciences	348 (25.11%)	147 (24.75%)		
- Medical & Life Sciences	179 (12.91%)	79 (13.30%)		
Monthly Allowance (RMB)	2,148 ± 615	2,154 ± 632	t = -0.20	0.841
Weekly MVPA (min/week)	194.2 ± 132.4	192.5 ± 128.5	t = 0.27	0.789
Walking Duration (min/week)	244.8 ± 143.1	246.1 ± 141.5	t = -0.18	0.857
Sleep Disturbance (JSS-4)	5.6 ± 3.1	5.6 ± 3.0	t = 0.16	0.873
Daily Screen Time (hours/day)	6.3 ± 2.2	6.3 ± 2.1	t = 0.22	0.826
Body Mass Index (kg/m²)	21.4 ± 3.2	21.4 ± 3.1	t = 0.12	0.905
Current Smoker, n (%)	120 (8.66%)	48 (8.08%)	χ^2 = 0.178	0.673
Active Alcohol Consumer, n (%)	242 (17.46%)	100 (16.84%)	χ^2 = 0.114	0.736
Academic Stress Score (1–10)	6.0 ± 1.9	6.0 ± 1.8	t = 0.35	0.726
Raw K10 Total Score (10–50)	17.8 ± 6.1	17.8 ± 5.9	t = 0.11	0.912

Categorical comparisons use descriptive Pearson χ^2 tests without continuity correction; they do not account for class dependence. Continuous-variable test statistics are as reported in the source analysis. Nonsignificant P values do not establish equivalence.
